# Feasibility and diagnostic accuracy of using brain attenuation changes on CT to estimate time of ischemic stroke onset

**DOI:** 10.1007/s00234-020-02591-w

**Published:** 2020-10-30

**Authors:** Grant Mair, Awad Alzahrani, Richard I. Lindley, Peter A. G. Sandercock, Joanna M. Wardlaw

**Affiliations:** 1 Edinburgh Imaging, and UK Dementia Research Institute at the University of Edinburgh and Centre for Clinical Brain Sciences, University of Edinburgh, Chancellor’s Building, 49 Little France Crescent, Edinburgh, EH16 4SB UK; 2 Centre for Clinical Brain Sciences, University of Edinburgh, Edinburgh, UK; 3 Westmead Applied Research Centre, University of Sydney, Sydney, Australia; 4 The George Institute for Global Health, Newtown, Australia

**Keywords:** Stroke, Ischemia, CT, Attenuation

## Abstract

**Purpose:**

CT attenuation of ischemic brain reduces with time after stroke onset. We aimed to quantify this relationship and test the feasibility and accuracy of estimating stroke onset time using only CT attenuation of visible ischemic lesions, *the CT-Clock Tool*.

**Methods:**

We selected CT scans with ischemic lesions representing a range of stroke-onset-to-scan times (elapsed time) from a well-defined stroke trial. We measured the attenuation of ischemic lesions and contralateral normal brain to derive attenuation ratio. We assigned scans to development (75%) or test (25%) datasets. We plotted the relationship between attenuation ratio and elapsed time in the development dataset and derived a best-fit curve. We calculated estimated time in the test dataset using only the attenuation ratio curve. We compared estimated time to elapsed time and derived absolute error for estimated time. We assessed area under the receiver operating characteristic (AUROC) curve for identifying scans ≤ 4.5 h elapsed time.

**Results:**

We included 342 scans from 200 patients (41% male, median age 83 years). Elapsed time range: 22 min to 36 days. Estimation errors were least at early elapsed times (r = 0.82, *p* < 0.0001): median absolute error was 23, 106, 1030 and 1933 min for scans acquired ≤ 3, > 3–9, > 9–30 and > 30 h from stroke onset, respectively. AUROC was high at 0.955.

**Conclusions:**

It is feasible to accurately estimate stroke onset time using simple attenuation measures of ischemic brain. Our method was most accurate 0–9 h from onset and may be useful for treatment eligibility assessment, especially where imaging resources are limited.

**Supplementary Information:**

The online version contains supplementary material available at 10.1007/s00234-020-02591-w.

## Introduction

In the first hours and days following the onset of an ischemic stroke, the CT attenuation of affected brain reduces with time. Within a few hours of symptom onset, ischemic lesions are often not visible to the naked eye. By 3–6 h, subtle changes such as loss of normal grey-white matter differentiation are appreciable but can be challenging to detect [1]. In the 12–48 h after onset, the ischemic lesion becomes easily identifiable through a combination of swelling and a more rapid decline in CT attenuation, such that the lesion appears darker than normal white matter.

Preclinical experimental data suggest that this progressive decline in CT attenuation represents worsening oedema, secondary to increases in both intra- and extra-cellular water. Importantly, CT scanners are calibrated to the attenuation coefficient of water, meaning that attenuation values are quantitative and ideal for measuring water uptake, even between scanners. Depending on the degree of impairment in cerebral blood flow, ischemic brain tissue suffers first cytotoxic (no net water shift), then ionic and finally vasogenic oedema (with greater degrees of net water uptake and swelling) [2, 3]. To the best of our knowledge, the relationship between the non-enhanced CT attenuation of ischemic brain and time has not been precisely evaluated in humans.

Up to 20% of ischemic strokes present with no known time of symptom onset [4], and in one large series, 36% of patients presented later than conventional eligibility time limits for treatment [5]. Recent randomized trials of both intravenous thrombolysis [6, 7] and mechanical thrombectomy [8, 9] that selected patients with imaging evidence of salvageable brain demonstrated treatment benefit for patients who present late or with an unknown time of symptom onset. However, these trials used imaging methods (contrast-enhanced CT perfusion imaging or MRI) that are not routinely available to all patients who present with stroke, especially outside of major stroke centres in developed nations [10, 11]. Ideally, imaging selection in this context would be as simple and widely applicable as possible so that the broadest range of patients can be considered for treatment without delay. For most patients worldwide who present acutely with ischemic stroke, only non-enhanced CT is likely to be available [10, 12].

The aims of our project were first to precisely quantify the relationship between non-enhanced CT attenuation of ischemic brain lesions and time using well-defined stroke trial data and second to test whether it is feasible to use only the CT attenuation of ischemic lesions to accurately estimate time of stroke onset for individual patients, the *CT-Clock Tool*.

## Materials and methods

### Study population

We used a subset of non-enhanced CT brain imaging from the Third International Stroke Trial (IST-3) for this retrospective feasibility analysis. IST-3 was a large (*n* = 3035) multi-centre randomized controlled trial testing IV alteplase given within 6 h of stroke symptom onset versus open control [13]. Ethical approval for IST-3 was granted by the Scotland A research ethics committee and local ethics committees. Consent was obtained for all patients. IST-3 was registered, Current Controlled Trials ISRCTN25765518. Enrolment, data collection and results for IST-3 have been published [13, 14]. Briefly, adult patients with acute stroke of any severity (assessed with the National Institutes of Health Stroke Scale, NIHSS) and no upper age limit were eligible if stroke onset time was clearly determined and treatment started within 6 h of onset, and baseline brain imaging had excluded haemorrhage and structural stroke mimics.

#### Case selection

IST-3 imaging protocols and the process for visual scan assessment by a central panel of experts have been described [15]. For the present analysis, we sought a subgroup of patients with non-enhanced CT brain imaging acquired both before treatment (baseline) and at follow-up (target follow-up imaging window was 24–48 h after stroke symptom onset, but this varied depending on clinical need and logistics; some patients also had repeat follow-up imaging at different time points), where at least one of these CT scans was thin slice (≤ 2.5 mm); i.e. we measured attenuation only on thin-slice CT. We used IST-3 central expert assessment of imaging to identify patients with obvious cerebral infarct at follow-up and to classify scans as with/without leukoaraiosis; central scan reads were not otherwise used in the present analysis. A neuroradiologist, specializing in stroke imaging confirmed the presence of a suitable ischemic lesion (clearly visible at follow-up and deemed large enough to measure) and excluded any scans with acute haemorrhage or degradation by imaging artefacts such that lesion measurement was likely to be inaccurate. We purposefully selected a comprehensive range of stroke-onset-to-scan times from the available baseline and follow-up thin-slice scans, as follows. For efficient spread of data points, we aimed to include a maximum of the following: one scan for each of the 360 min from 0 to 6 h (up to 360); one scan for every 10 min window from 6 to 12 h (up to 36); one scan for every 30 min window from 12 to 48 h (up to 72); one scan per hour from days 3–7 (up to 120); and any scans from later time points (i.e. up to a maximum of 588 scans). To help reach these targets, we allowed baseline and follow-up scans from the same patient to be included. If multiple scans were available for a given time point, we randomly selected only one. Note, we did not intend this population to be representative of routine clinical practice, we selected patients purely on the basis of measurable ischemic lesions across the desired time range in order to best quantify the CT attenuation-time relationship in ischemic stroke.

### Measuring CT attenuation

**Fig. 1:**
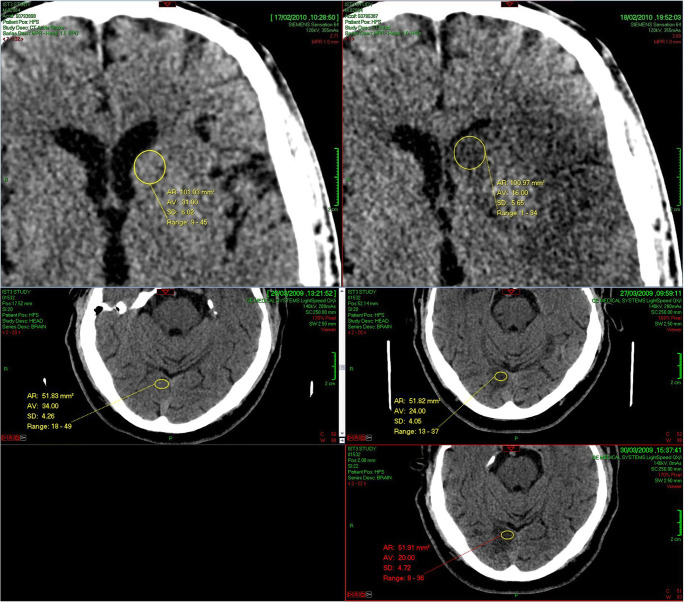
ROI placement within the same ischemic lesions on sequential thin-slice CT scans for two different patients. **Note**: Upper panel: Two sequential scan measurements from the same patient, left image at 175 min from stroke symptom onset, right image at 36 h from onset. Lower panels: Three sequential scan measurements from the same patient, left image at 120 min, middle right image at 22 h, and lower right image at 100 h from stroke onset

After comparing different methods (see *Supplementary Material*), we assessed CT attenuation changes of brain by application of manual regions of interest (ROI) within ischemic lesions and contralateral normal brain mirrored on sagittal midline. We thus derived an attenuation ratio for each scan as ischemic brain/normal brain. We used Carestream PACS (v11, Carestream Health Inc. Rochester, NY, USA) to view all CTs using standard brain (centre 35 HU (Hounsfield Units), width 70 HU) and/or narrowed ‘stroke’ (centre 25–35 HU, width 30–40 HU) window settings, modified according to user preference. Zoomed images were used to ensure accurate placement of ROIs. The same stroke specialist neuroradiologist applied all ROIs using standard Carestream Oval ROI Measurement Tool and where possible placed a 100 mm^2^ area round or ovoid ROI. We used smaller ROIs if required (e.g. to avoid CSF spaces or focal calcification), but paired measurements (e.g. between the ischemic lesion and normal brain or between baseline and follow-up scans in the same patient) always used area matched ROIs (Fig. 1). If ischemic lesions were not clearly visible on baseline scans, we used follow-up imaging (viewed in parallel and co-registered using the Carestream *Automatic Registration* tool) to guide ROI placement. ROI placement was conducted blind to time from stroke onset. When an ischemic lesion or lesions were visible, we sampled the area with the lowest attenuation based on visual assessment and ROI placement in as many candidate areas as needed for each scan. We sampled both white matter and grey matter with no preference. We recorded the lowest mean attenuation within sampled ROIs and recorded the standard deviation (SD) of the attenuation measurement for each ischemic lesion. Thus, we tested the precision of ROI measurements of ischemic lesions and compared intra- and inter-lesional measurement variance. We assessed relationships between measurement precision of ischemic lesions, time from stroke onset and background leukoaraiosis of brain as potential confounders.

#### Testing reader reliability

We performed reader-reliability analyses using a random subsample of our study population assessed separately. A second reader, masked to all clinical and imaging data (including previous ROI placement and results) and with limited prior experience assessing ischemic stroke lesions on CT, was trained using the same method described above and independently scored 92 scans from 47 patients. We assessed inter-rater agreement using intraclass correlation coefficient (ICC) estimates (and their 95% confidence intervals, 95%CI) based on a single rating, absolute agreement, two-way random effects model [16]. Second reader results were not otherwise used.

### Estimating time from stroke onset

We recorded the time and date of each CT scan from the DICOM headers during ROI application. We then derived a new variable *elapsed time*, as the difference between CT scan time/date and recorded symptom onset time/date. Blind to all attenuation measurements and all clinical data except *elapsed time*, we divided our cohort into development (75%) and test (25%) datasets. To ensure that *elapsed time* was represented equally in both development and test datasets, we made minor reallocations as necessary.

We used the development dataset to quantify the relationship between change in ischemic lesion attenuation and time. We plotted attenuation measures against *elapsed time* and derived a best-fit logarithmic curve. We inverted the function of this curve to create a formula for estimating time from symptom-onset-to-scan (*estimated time*) using only attenuation measures. We then applied our formula to each scan in the test dataset to derive *estimated time*.

### Validation of time estimation results

We compared *estimated time* and *elapsed time* in the test dataset to quantify time estimation errors. We sought trends for over- or under-estimation by subtracting *estimated time* from *elapsed time* and assessed whether differences were positive (under-estimate) or negative (over-estimate). We used absolute error for all validation assessment; i.e. we used the magnitude differences between *estimated* and *elapsed time* by converting negative time differences to positive so that all values were greater than zero. We also derived absolute percentage error (absolute error/*elapsed time* × 100). We correlated absolute error with *elapsed time* and with attenuation measures. We selected subgroups of *elapsed time*, chosen to present similar case numbers at clinically relevant cut points (i.e. early, delayed, late and extremely late) and assessed the relationship between error and *elapsed time*. Finally, with *elapsed time* as the reference standard and our attenuation measurements as the index test, we performed a receiver operating characteristic (ROC) curve analysis on the whole dataset to test the theoretical discriminative ability of our *CT Clock Tool* for determining intravenous alteplase eligibility according to the European licensing limit of 4.5 h.

### Statistical analyses

We present normally distributed data using mean (SD) and non-parametric data as median (inter-quartile range, IQR). For comparisons of normally distributed or dichotomous data, we used independent sample *t*-tests. We used independent sample Mann-Whitney *U* or Kruskal-Wallis tests for comparisons and Spearman’s Rho for correlations of non-parametrically distributed data. We used Bland-Altman statistics for assessing relationships between *estimated time* errors and *elapsed time* [17]. For ROC analysis, we used Youden’s index maximum value to identify the attenuation measurement cut-off with the optimum balance of sensitivity and specificity and estimated area under the ROC curve.

We performed all analyses using IBM SPSS Statistics software, versions 20 and 22 (IBM Corporation, Armonk, NY, USA). We considered a *p* value < 0.05 significant.

## Results

### Study population

**Fig. 2:**
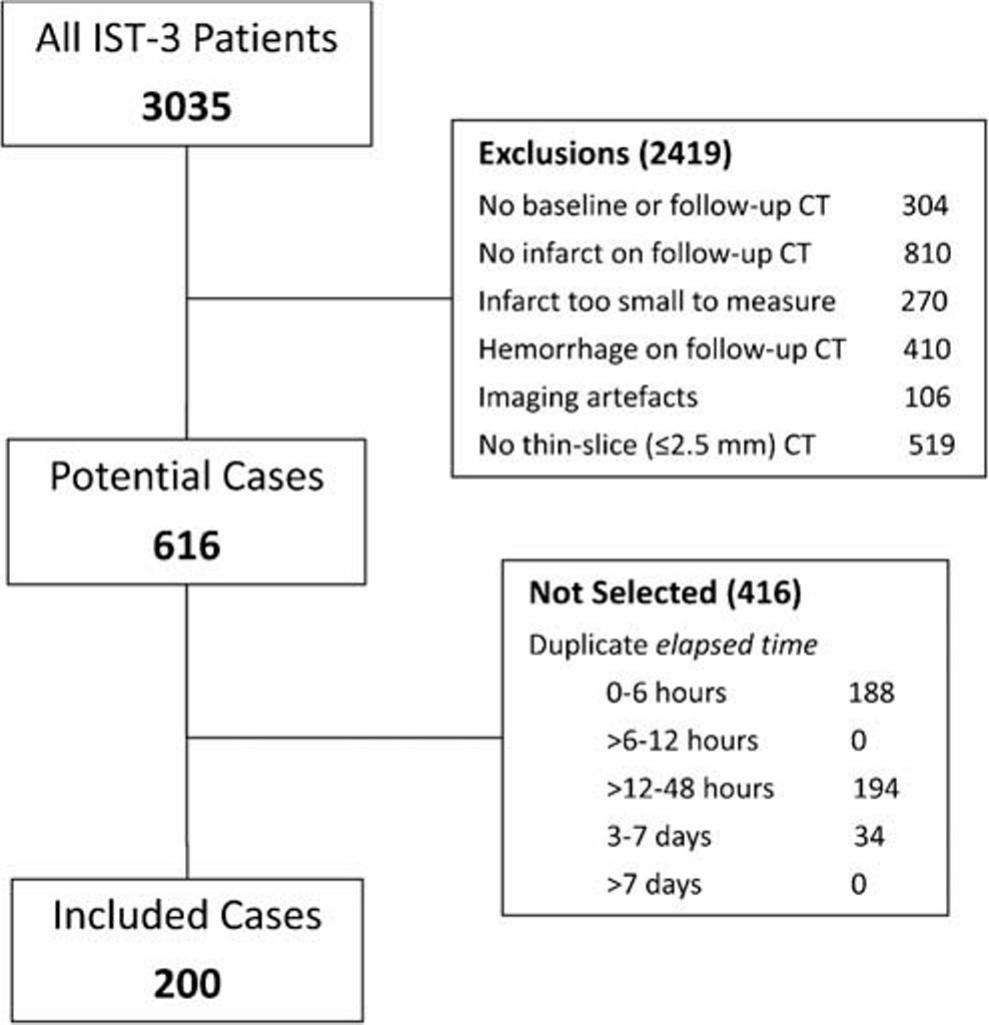
Flowchart for patient selection

We selected and assessed 342 CT scans from 200 patients (Fig. 2). Most patients (*n* = 105, 52.5%) contributed 2 scans, 77 patients (38.5%) contributed 1 scan, 17 patients (8.5%) contributed 3 scans, and 1 patient (0.5%) contributed 4 scans. Nearly half of all scans (*n* = 162, 47.4%) were acquired < 6 h of stroke onset. Due to IST-3 design, few scans were available 6–12 h (*n* = 4, 1.2%) or 12–24 h (*n* = 14, 4.1%) after stroke onset. Most remaining scans (*n* = 114, 33.3%) occurred 24–48 h from stroke onset, with 42 (12.3%) acquired between 3 and 7 days, and only 6 (1.8%) acquired beyond 7 days. Among all scans, *elapsed time* ranged from 22 min to 865 h (36 days), median 16.7 h, IQR 2.6 to 30.8 h.

Of the 200 patients included, 81 (40.5%) were male; the median age was 83 years, IQR 74–87 years; baseline stroke severity was moderate-severe with median NIHSS 17, IQR 11–21; and 93 patients (46.5%) were allocated to alteplase. There were no significant differences between patients in the development (*n* = 149) and test (*n* = 51) datasets, Table 1.

**Table 1:** Comparison of development and test datasets

Variable	Development dataset (75%)	Test dataset (25%)	*p* value for difference
Patient group comparisons (*n* = 200)	n = 149	*n* = 51	
Age (years)	84 (77–87)	81 (68–86)	0.065
Male sex	60 (40.3%)	21 (41.2%)	0.910
NIHSS	17 (11–21)	17 (10–22)	0.768
Allocated to alteplase	70 (47.0%)	23 (45.1%)	0.817
CT Scan characteristic comparisons (*n* = 342)	n = 256	*n* = 86	
Elapsed time (hours)	21.1 (2.6–30.7)	15.4 (2.5–32.2)	0.944
Ischemic lesion attenuation (HU)	22.6 (6.0)	22.5 (6.4)	0.842
SD of individual lesion measurements (HU)	5.3 (1.3)	5.5 (1.4)	0.208
Contralateral normal tissue attenuation (HU)	30.8 (3.4)	30.7 (3.5)	0.960
Attenuation ratio	0.73 (0.18)	0.73 (0.18)	0.805
Presence of leukoaraiosis†	132 (51.6%)	39 (46.4%)	0.416

Results are mean (standard deviation = SD), median (inter-quartile range) or *n* (percentage) as appropriate. 200 patients provided 342 CT brain scans for analysis. Elapsed time = time from stroke symptom onset to scan. HU = Hounsfield Units. †Leukoaraiosis data missing for 2 test dataset scans

### Measuring CT attenuation changes

From 342 CT scans assessed in total, the mean ischemic lesion attenuation was 22.6 (SD 6.1) HU, compared with a mean attenuation of 30.8 (SD 3.5) HU in contralateral normal brain. Thus, the mean attenuation ratio for all scans was 0.73. Precision was consistent for intra- and inter-lesional measurement, the mean SD of individual lesions was 5.4 HU compared to 6.1 HU for the entire group. The SD for individual lesion measurements was not different for scans with versus without background leukoaraiosis (*p* = 0.065), or for scans performed before or after 4.5 h (*p* = 0.537), and there was no correlation between lesion SD and elapsed time (*p* = 0.352), or lesion SD and attenuation (*p* = 0.05). There were no significant differences of these measured CT characteristics or *elapsed time* between the development (*n* = 256) and test (*n* = 86) datasets (Table 1).

Inter-rater agreement for the assessment of ischemic lesions, contralateral normal brain and attenuation ratio was ICC (95%CI) = 0.89 (0.82–0.93), 0.55 (0.39–0.68) and 0.85 (0.75–0.91), respectively.

### Estimating time from stroke onset

**Fig. 3:**
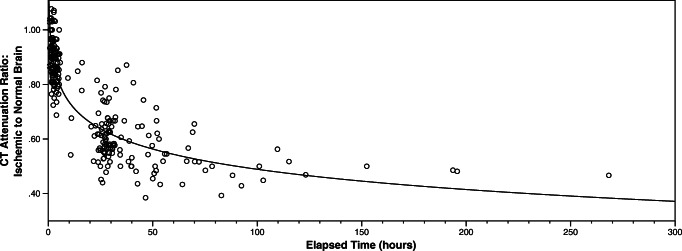
Best-fit logarithmic function for attenuation ratio versus elapsed time in the development dataset (*n* = 256)

Figure 3 demonstrates the best-fit logarithmic curve (*R*^2^ = 0.768) derived by plotting attenuation ratio against *elapsed time* for the 256 scans in the development dataset. A few (*n* = 10) very early scans have an attenuation ratio greater than 1.0; i.e. CT attenuation in the affected brain is greater than the CT attenuation of contralateral normal brain. Attenuation ratio drops thereafter, and according to the best-fit curve measures 0.86, 0.82 and 0.79 at 3, 4.5 and 6 h *elapsed time*, respectively.

**Fig. 4:**
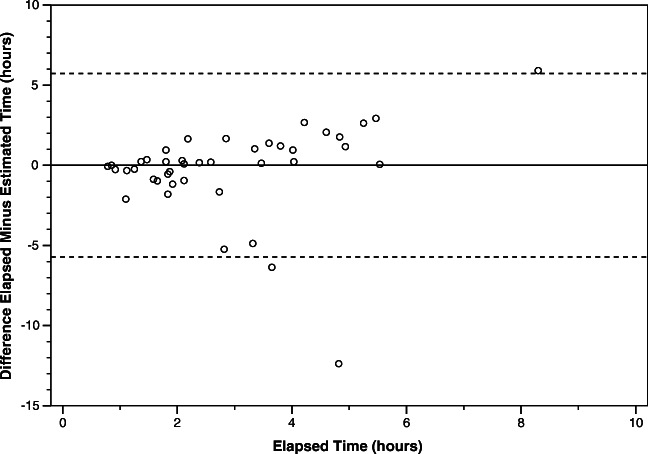
Bland-Altman analysis of error in estimated time results. **Note:** Difference between *elapsed* and *estimated time* (horizontal solid line) in the test dataset. For clarity of presentation, only includes results from the most clinically relevant early (≤ 3 h) and delayed (> 3 to 9 h) *elapsed time* subgroups. Mean − 0.2 h, SD 2.9, *n* = 42. Dotted lines represent ± 2 SD from mean

In the test dataset (*n* = 86), differences between *estimated* and *elapsed time* were well balanced but with a slight trend toward under-estimation (45 positive versus 41 negative differences). Absolute error ranged from < 1 min to 247 h, with a median of 315 min. The median absolute percentage error was 51% (IQR 25–72%). Absolute error was highly correlated with elapsed time (r = 0.82, *p* < 0.000001) and with attenuation measures (ischemic lesion attenuation r = −0.83, *p* < 0.000001, attenuation ratio *r* = − 0.86, *p* < 0.000001) such that errors were smallest for scans with early or less hypoattenuating ischemic lesions (Fig. 4 and Table 2). We identified the following *elapsed time* subgroups in the test dataset: early (≤ 3 h, *n* = 25), delayed (> 3 to 9 h, *n* = 17), late (> 9 to 30 h, *n* = 20), extremely late (> 30 h, *n* = 24) (Table 2). The median absolute error was 23 min for early, 106 min for delayed, 1030 min for late and 1933 min for extremely late scans (*p* value for difference < 0.0001). A similar trend was observed for absolute percentage errors.

**Table 2:** Validation of *estimated time* results in the test dataset

Group	*Elapsed time* median (IQR), range	*Estimated time* median (IQR), range	Over- and under-estimates (n)	Absolute error median (IQR)	Absolute percentage error median (IQR)
All (*n* = 86)	924 (152–1932), 47–51,914	524 (146–3153), 32–37,069	41 over 45 under	315 (57–1486)	51 (25–72%)
≤ 3 h (*n* = 25)	110 (78–129), 47–171	122 (69–170), 32–483	14 over 11 under	23* (14–85)	29 (14–60%)
> 3 to 9 h (*n* = 17)	253 (217–305), 199–498	184 (148–278), 93–1032	3 over 14 under	106* (60–234)	38 (24–67%)
> 9 to 30 h (*n* = 20)	1567 (1452–1689), 873–1793	1477 (672–4467), 367–8778	10 over 10 under	1030* (597–2947)	65 (48–186%)
> 30 h (*n* = 24)	2950 (2209–5725), 1810–51,914	3661 (1994–6360), 701–37,070	14 over 10 under	1933* (705–3976)	52 (29–66%)

All results are in minutes, unless otherwise stated. IQR = interquartile range. Subgroups are based on *elapsed time*. *4 groups significantly different, *p* < 0.0001

**Fig. 5:**
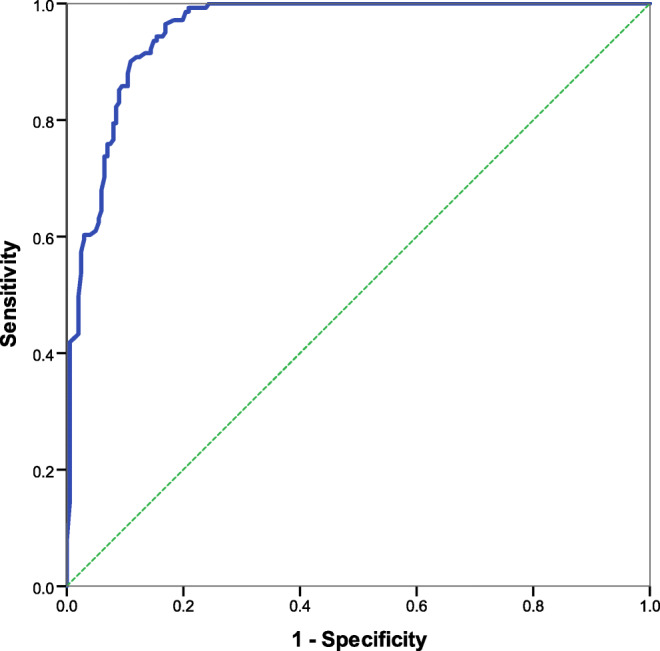
ROC analysis testing the expected discriminative ability of attenuation ratio to determine intravenous alteplase eligibility as per 4.5 h European licensing limit, *n* = 342. **Note:** Area under curve = 0.955

ROC analysis of all 342 scans suggested that an attenuation ratio of 0.753 (Youden’s index = 0.80) would provide the optimum test sensitivity and specificity (96.5% and 83.1%, respectively), for correctly classifying scans as before or after the 4.5 h European licensing limit for intravenous alteplase. The area under the curve for ROC analysis was high at 0.955 (95% confidence interval 0.936–0.975) (Fig. 5).

## Discussion

In this feasibility analysis, we provide evidence that it is possible, with high accuracy and reasonable precision, to estimate time from ischemic stroke onset using simple CT brain attenuation measurements alone, particularly during the early, most clinically relevant period. By closely mapping the attenuation changes of ischemic brain with time in a well-defined stroke trial dataset, we have derived a formula for understanding the time-attenuation relationship in humans. We used the ratio of manually measured CT attenuation in ischemic brain relative to contralateral normal brain. Our *CT Clock Tool* can provide specific estimates of stroke onset time for individual patients where ischemic lesions are visible on NECT without the need for advanced imaging or specialist software.

### Agreement, error and accuracy of the CT Clock Tool

We found that time of stroke onset estimates were accurate relative to true *elapsed time,* especially < 9 h from stroke onset. The accuracy of our estimates was time dependent, estimates for early scans (0–3 h) were more accurate (median error ± 23 min) than estimates for delayed scans (± 106 min for scans performed 3–9 h from stroke onset). The difference between *estimated time* and *elapsed time* was strongly positively correlated across the sampled time range; the closer patients were to their stroke onset time, the more accurate the prediction. Similarly, time estimation errors were strongly negatively correlated with lesion attenuation; i.e. the more obvious (darker) the lesion, the greater the error. These associations are clinically reassuring since methods to determine treatment eligibility for patients with ischemic stroke are most needed in the first hours after stroke onset, when lesions are not overtly visible. The European licensing limit for intravenous alteplase is 4.5 h and patients who wake up with stroke symptoms are likely to be 6–9 h since last known to be well (i.e. before they went to sleep). Therefore, the clinical challenge is often to determine which patients are within and which are outside the 0–4.5 h treatment window for alteplase. Minnerup and colleagues estimated that 11.5% water uptake on non-enhanced CT (derived from attenuation change) was the optimal cut off to classify patients within the 4.5 h European licensing limit with high sensitivity (91–99%) and specificity (78–91%), but used contrast-enhanced CT perfusion imaging to first identify ischemic lesions [18, 19]. On ROC analysis, the estimated optimum test sensitivity and specificity of our *CT Clock Tool* compared well with the Minnerup method at 97% and 83%, respectively. The true diagnostic accuracy of our *CT Clock Tool* for identifying patients who are eligible for alteplase will require further testing in a clinically representative cohort and has the advantage of not requiring perfusion imaging for guidance.

### Precision and reliability of CT attenuation measurements

Given the deliberately wide *elapsed time* range included in our analysis, we expected and found greater variability of attenuation measurements for ischemic brain tissue compared to equivalent measurements of contralateral normal brain tissue (at the group level SD 6.1 versus 3.5, respectively). We might also have expected this variability in ischemic lesion measurement to increase with *elapsed time* or with the natural progression of lesions, but we found no evidence of this. In other words, the extent of variability was not affected by time from stroke onset. Similarly, we found no difference in lesion measurement variability between those with and those without leukoaraiosis, a common coexistent finding among patients with stroke. We also found measurement variance within individual ischemic lesions comparable to the variance between different ischemic lesions.

Reader reliability for our method was excellent. We found high levels of agreement for lesion measurement between experienced and inexperienced readers (ICC 0.89). Interestingly, agreement was slightly less for attenuation measurement of the contralateral normal brain (ICC 0.55), but this did not significantly negatively impact agreement for the calculated attenuation ratio (ICC 0.85) thus *estimated time* is unlikely to be greatly different between readers.

Such consistency and reliability of lesion measurement are reassuring for the potential future clinical application of our *CT Clock Tool*. On the other hand, we did observe greater error for time estimation when scans were performed late versus early. This may reflect fewer attenuation measures in our study at these later times but is also likely indicative of the natural heterogeneity of lesion progression. Rocha and Jovin describe *fast and slow progressors* among patients with proximal large vessel obstruction being considered for thrombectomy and suggest that variability in individual patient trajectories relates to differences in their collateral circulation [20]. Those with good collateral supply are likely to sustain viable brain tissue for longer and this concept is backed by pre-clinical evidence [21]. In this context, it is possible that some of the apparent discrepancies between our *CT Clock Tool* and *elapsed time* reflect real CT measurable inter-patient differences, rather than error in our technique. We might be estimating time since infarct rather than time since stroke symptom onset for patients with good collateral supply, but this would require further testing. Kucinski and colleagues demonstrated in a small group (25) of patients with early ischemic stroke (< 5.4 h) that apparent diffusion coefficient values (ADC, a component of diffusion weighted MRI and often considered to represent infarct) of ischemic lesions correlate with measurable non-enhanced CT attenuation changes, albeit weakly [22]. In a small cohort of patients (41) undergoing thrombectomy, Mokin and colleagues similarly estimated that attenuation ratios of 0.94–0.96 most consistently predicted tissue death [23]. These attenuation ratios fit with the precipitous decline in attenuation demonstrated during the first hour after stroke onset in our study (Fig. 3). Ultimately, patient-specific lesion measurements are likely to be more powerful predictors of brain tissue viability and thus favourable treatment response than standard measures of elapsed time in stroke and might be used to offer treatment to patients who present late. An interesting and potentially novel observation in our analysis was that the attenuation of affected brain briefly *increased* in the very early stages after ischemic stroke onset immediately prior to the described rapid decline. This may reflect increased regional blood volume, which is a recognized perfusion-imaging feature of ischemia rather than infarct and likely a physiological attempt to maximize nutrient delivery to the injured but still viable brain tissue. Whether measuring this increase in CT attenuation could provide a meaningful assessment of brain tissue viability remains untested. Associations between specific lesion measurements thought to indicate tissue viability and improved clinical outcome following treatment, even at extended *elapsed times*, are beginning to emerge [6–9]. However, all of these methods require the use of additional or advanced imaging in the acute setting, which for the purpose of broad clinical applicability we were keen to avoid.

### Strengths and limitations of the present work and future plans

We used the location of infarcts on follow-up imaging to measure attenuation in very early ischemic lesions on baseline imaging that were not yet visible to the human eye (generally within the first hour) so that these too could be included in order to achieve a more complete understanding of the time-attenuation relationship. This information would not be available in a prospective analysis without the use of complementary advanced imaging to guide ROI placement. So while our *CT Clock Tool* can be used without the need for MRI or contrast-enhanced CT imaging (angiographic collateral scoring or perfusion imaging) as others have done, we still need to develop the most appropriate method for applying the technique when lesions are not easily seen. For example, determining when an ischemic lesion is likely to be present but not visible versus not present. But for those with visible lesions, we wanted to develop our method using manually placed ROIs because this approach requires no specialist equipment or software and is therefore readily replicable.

Developing a system that can make accurate estimates of stroke onset time using only CT attenuation should ensure that our *CT Clock Tool* can be widely applicable, even where resources are limited, i.e. in any centre that can offer non-enhanced CT to patients with symptoms of stroke. Our precision, accuracy and inter-reader reliability results support the robustness of this method. However, we are also exploring automated computational methods for the application of our *CT Clock Tool* since assisted lesion detection may be the most suitable method for applying our technique in many circumstances and may improve the reliability of lesion measurements, but these assertions are currently unproven and such methods are not yet widely available.

Our analysis includes a very broad range of *elapsed times*, deliberately selected to be well-beyond recognized treatment windows for ischemic stroke. This design was to ensure our assessment of the relationship between attenuation and *elapsed time* was comprehensive. Best-fit curves are likely to benefit from more complete data, and our method also needs to include patients who present very late to appropriately classify their scans. We acknowledge this broad time range extends beyond the period for acute therapy. Similarly, we selected non-conventional subgroups for *elapsed time* based on the relatively small numbers available in our test dataset so that our *early, delayed, late and extremely late* subgroups were balanced while remaining clinically relevant. As described above, up to 9 h from symptom onset is likely to be most relevant for patients with wake-up stroke and for whom our *CT Clock Tool* may be most helpful. We anticipate exploring more conventional *elapsed time* windows (e.g. ≤ 3 and ≤ 4.5 h) as additional data are included in our model. We also expect that given the strong relationship between estimation error and *elapsed time*, that error is reduced for patients in the critical early first half of our 3–9 h subgroup.

After the initial oedema that predominates the decrease in CT attenuation of early brain ischemia and is utilized in our study, more complex changes such as influx of protein-rich fluids, petechial/capillary haemorrhage and compression of tissues might act to conversely *increase* the CT attenuation of affected brain at later time-points. Although we actively excluded visible haemorrhage and sought scans with obvious lesions at follow-up, these imperceptible processes could not be completely excluded and may partly explain greater error at later time-points. Similarly, fogging of ischemic lesions on brain CT is a transient phenomenon that can occur 2–3 weeks after stroke onset where the attenuation of lesions briefly appears to normalize before gliosis becomes readily apparent [24, 25]. This pseudonormalization of ischemic lesions will not affect our measurements or time estimates during the critical first hours after stroke onset but could have affected our understanding of the time-attenuation relationship at extremely delayed times. However, by only selecting cases with obvious lesions on follow-up imaging, we should have minimized this effect.

Potential sources of bias in our work are the use of only one experienced scan reader for the primary analysis, use of multiple scans from the same patient, selection of patients based on scan findings and time from stroke onset rather than representative clinical features and not reaching our aspirational target of 588 scans to cover the entire predefined time range using IST-3 data alone. We plan to incorporate CT imaging data from other sources, and we will continue to add new cases to our development dataset until we meet the *elapsed time* distribution targets described in methods. We also plan to validate our technique in a larger representative dataset, to provide human reader-reliability testing across a greater and more representative range of reader abilities, and finally to prospectively test the safety and applicability of our *CT Clock Tool* in clinical populations. Thereby, we hope to maximize the real-world applicability of the data used to calculate *estimated time*.

## Conclusions

We have quantified the relationship between non-enhanced CT attenuation of ischemic brain lesions and time and developed an easily replicable method for estimating time from stroke symptom onset using only simple attenuation measurements of visible lesions, the *CT Clock Tool*. Our initial feasibility testing indicates reasonable precision, accuracy and reliability of the method. With further development and testing, our technique may offer a very quick and widely applicable method for helping determine treatment eligibility in ischemic stroke.

## Supplementary Information

ESM 1 (DOCX 403 kb)

## Data Availability

IST-3 data are available on request: https://datashare.is.ed.ac.uk/handle/10283/1931
