# Supplementary material for: Feasibility and diagnostic accuracy of using brain attenuation changes on CT to estimate time of ischemic stroke onset
Source: Neuroradiology. 2020 Oct 30;63(6):869–78. doi: 10.1007/s00234-020-02591-w (PMC8128810; doi:10.1007/s00234-020-02591-w)
Supplement: Supplementary file 1 — (DOCX 403 kb) [file 234_2020_2591_MOESM1_ESM.docx]

**Feasibility and diagnostic accuracy of using brain attenuation changes on CT to estimate time of ischemic stroke onset**

**Supplementary Material**

**Development and testing of our CT attenuation measurement method for time estimation**

We initially tested different methods for determining attenuation change in ischemic brain tissue that might limit the effect of variability caused by between-scanner and between-patient differences such as poor CT scanner calibration or background leukoaraiosis of brain.

*Development of attenuation measurement method:*

For a subset comprised of the first 120 scans, we compared absolute tissue attenuation (Hounsfield Units, HU) derived solely from within the ischemic lesion to HU values corrected for scanner calibration errors by air offset (air surrounding the patient should always be exactly -1000 HU, e.g. if air measured -995 HU, we would adjust attenuation measurements of brain by +5 HU for that scan) and/or normalised to one of: contralateral normal brain mirrored on sagittal midline (i.e. including equivalent volumes of grey and white matter), contralateral thalamus, contralateral cortex, contralateral white matter (Figure). For each ROI attenuation pair, an attenuation (HU) ratio was derived as ischemic lesion attenuation/other. We plotted corrected/uncorrected HU values and attenuation ratios against known time of symptom onset and derived best-fit curves for each. We used R^2^ values for curve estimation to determine which method of normalising/calibrating brain attenuation change (and which type of curve) produced the least variability (greatest R^2^ value) and selected this as our preferred method.

*Testing of attenuation measurement method:*

Based on this testing, we selected ischemic lesion to contralateral normal brain attenuation ratio (without air calibration) as our preferred method, and we selected a logarithmic curve for fitting data (best fit R^2^ = 0.71), Table.

We then used only our preferred method for assessing brain attenuation change in all subsequent scans for the remainder of the study population and in all analyses.

**Table.** Comparison of CT attenuation measurement techniques and best-fit curve type, n=120.

| **CT attenuation measurement technique** | **With air calibration** | **R-squared (R^2^)** |
| --- | --- | --- |
| Absolute attenuation of lesion | No | 0.59 |
|  | Yes | 0.50 |
| Ischemic lesion to contralateral normal brain attenuation ratio | No | 0.71 |
|  | Yes | 0.54 |
| Ischemic lesion to contralateral thalamus attenuation ratio | No | 0.61 |
|  | Yes | 0.48 |
| Ischemic lesion to contralateral white matter attenuation ratio | No | 0.66 |
|  | Yes | 0.45 |
| Ischemic lesion to contralateral cortical grey matter attenuation ratio | No | 0.58 |
|  | Yes | 0.47 |
| **Best-fit curve type** | | |
| Logarithmic | - | 0.71 |
| Inverse | - | 0.30 |
| Exponential | - | 0.53 |
| Logistic | - | 0.51 |

**Note:** Best-fit curve type results are presented here only for the measurement technique that provided the best results, *Ischaemic lesion to contralateral*.

**Figure.** Region of interested sampling locations in subgroup (n=120) to test attenuation correction with air offset and normalisation using different locations within contralateral normal brain.


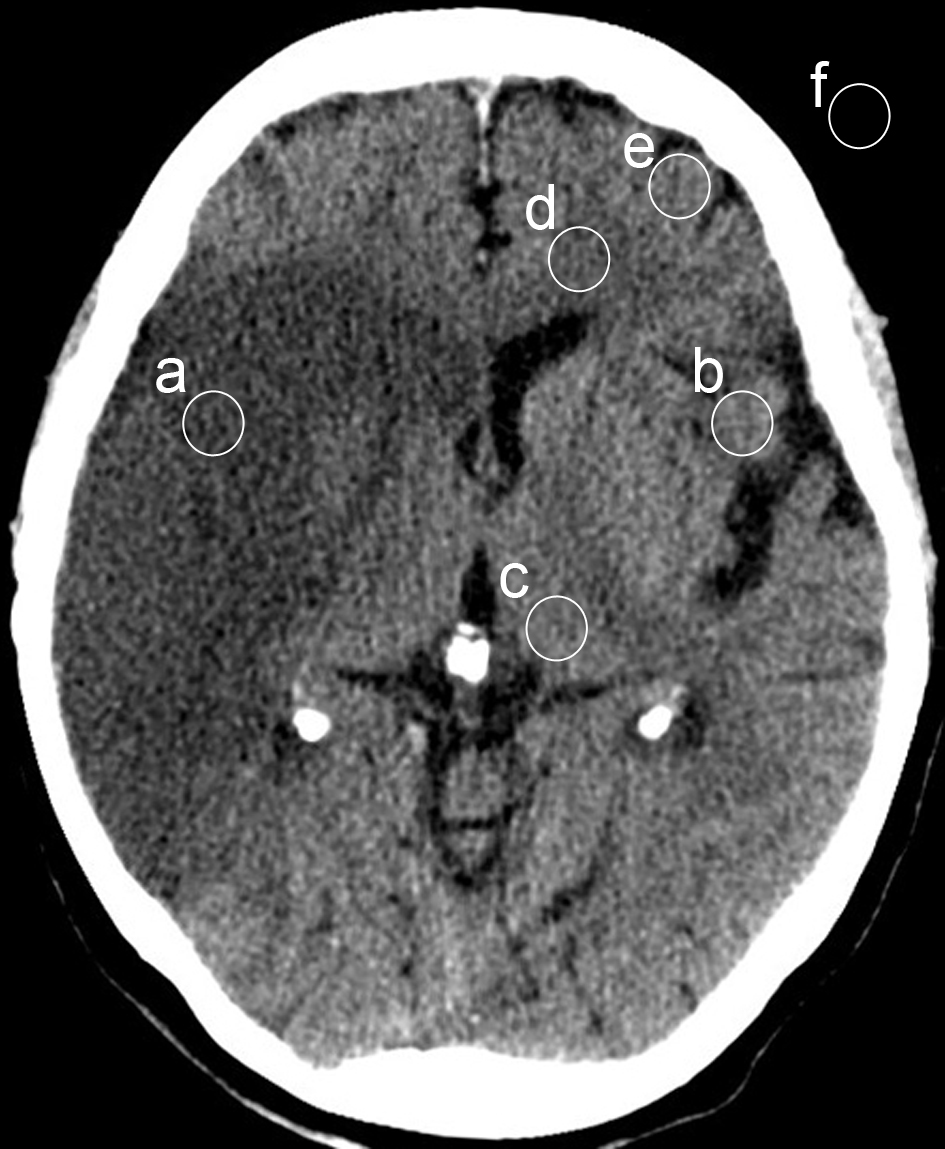


**Note**: Sampling locations are: a) ischemic lesion, b) contralateral equivalent normal brain mirrored on sagittal midline, c) contralateral thalamus, d) contralateral white matter, e) contralateral cortex, and f) air surrounding the patient.
